# Associations of Functional Movement Screen Scores and Joint Stability Tests with Non-Contact Lower-Limb Injury Burden in Collegiate Athletes

**DOI:** 10.3390/jcm15166466

**Published:** 2026-08-21

**Authors:** Adam Eckart, Pragya Sharma-Ghimire

**Affiliations:** Department of Health and Human Performance, Kean University, 1000 Morris Avenue, Union Township, NJ 07083, USA; pragya.sharma@kean.edu

**Keywords:** injury risk, functional movement screen, joint instability tests

## Abstract

**Background:** The ability of preseason screening to identify athletes at risk for non-contact lower-limb injury remains uncertain. **Purpose:** To determine whether knee and ankle joint tests, body mass index (BMI), prior lower-limb injury, and Functional Movement Screen (FMS) scores were associated with injury and improved discrimination. **Methods:** Prospectively collected data from 381 collegiate athletes across 11 sports were retrospectively analyzed. Preseason assessments included BMI, injury history, FMS composite and subtest scores, and knee and ankle special tests summarized regionally. The primary outcome was two or more non-contact lower-limb injuries during the subsequent season; at least one injury was secondary. Hierarchical multivariable logistic regression, sex-stratified and sensitivity analyses, and cross-validated ridge logistic regression were performed. **Results:** Eighty athletes (21.0%) sustained at least one injury and 41 (10.8%) sustained two or more. Prior injury was associated with ≥2 injuries in Models 1 and 2 (odds ratios = 2.03 and 2.00, respectively) and remained the most consistent correlate across sensitivity analyses. Knee and ankle test findings and FMS composite scores were not consistently associated with injury in primary analyses. Exploratory subgroup associations included female BMI, In-Line Lunge, female Deep Squat, and male ankle instability without prior injury. For the primary outcome, apparent AUC was 0.621–0.649 and cross-validated AUC was 0.535–0.562; adding FMS variables did not improve cross-validated discrimination. **Conclusions:** Prior injury history was the most consistent risk marker, but the preseason battery showed limited generalizable predictive value. FMS and joint-test measures should not be used alone to predict individual injury risk.

## 1. Introduction

Musculoskeletal injuries (MSIs) during sports participation are common and often result in lost match and training time [1,2,3,4]. Exposure rates vary substantially by sport, competition level, and whether the sport is contact or non-contact [1,2,3,4]. Individual-level characteristics also contribute to differential injury rates and the incidence of various injury types [5]. Individual-level factors include injury history, training experience, age, sex, body composition, joint architecture, sleep, and acute-to-chronic workload ratios [5]. Despite substantial work on injury risk, predicting first-time and recurrent injury with high precision remains elusive [1,2,3,4,5]. Field-expedient movement screens, such as the Functional Movement Screen (FMS), despite their popularity, have shown limited utility in predicting injuries, primarily due to issues with test validity [5,6,7,8]. Several criticisms are levied against tests like the FMS, as they do not consider essential injury factors such as age, sex, body composition, experience, sport, force, or kinematic measures. Moreover, the underlying assumptions of these tests are problematic, as they generalize biomechanical patterns and motor function from a limited set of movements to complex, dynamic athletic movements, during which injuries often occur [5].

Recent research using multifactorial injury risk models that include individual-level predictors has shown promising predictive ability [9,10]. In one case-control study, measures of anterior-posterior knee laxity, posterior knee stiffness, navicular drop, and standing quadriceps angle were significantly associated with first-time non-contact anterior cruciate ligament (ACL) injury in males [9]. In females, parental ACL injury, anterior-posterior knee laxity, and body mass index (BMI) were significantly associated. In another study, a combination of predictors, including knee medial displacement, BMI, ankle dorsiflexion with the knee extended, single-leg counter-movement jump, passive hip internal rotation, frontal plane projection angle, and the tuck jump, was most predictive of lower-limb injuries in youth male soccer players. The injury model demonstrated reasonable specificity but poor sensitivity [10].

Although several studies suggest that the FMS poorly predicts sports injuries on its own, some evidence indicates that models using FMS subtests, rather than just the composite score, combined with various performance tests or other individual-level predictors, may improve model performance [11]. For example, a study of 167 Division 1 collegiate athletes found that female sex, older age, lower BMI, and the in-line lunge FMS test were significantly associated with non-contact MSIs [12]. This may be because FMS subtests capture region-specific dysfunction, including sport-specific limb or movement-pattern asymmetries [5,11,13].

Joint laxity has received substantial attention as a predictor of MSIs [14,15]. Generalized joint hypermobility (GJH), often assessed with the Beighton score, is a significant risk factor for specific injuries, such as ACL rupture or lateral ankle sprain [15]. Although the strength of this association varies across populations, GJH is considered a marker of underlying connective tissue properties that may predispose athletes to injury [14]. Notably, a history of joint injury remains one of the most consistent and powerful predictors of future injury, reflecting the cumulative effects of tissue compromise and altered neuromuscular control [16]. Special joint tests, such as the Lachman test for ACL laxity, the anterior drawer test for ankle instability, and shoulder/patellar apprehension tests, offer a direct clinical means of identifying residual laxity or instability after injury. Although these special tests are conventionally used for diagnosis, growing evidence suggests that the anatomical risk factors they reveal predict recurrent injuries [16,17]. Therefore, integrating GJH, prior injury, and joint-specific laxity into multifactorial injury risk models provides a rationale for incorporating special joint tests into preseason screening, particularly for non-contact lower-limb injuries.

Despite the recognized roles of generalized joint hypermobility, prior injury, and residual laxity in increasing athletes’ risk of musculoskeletal injury, few studies have integrated these factors into a comprehensive risk model. Moreover, most existing screening approaches, such as the Functional Movement Screen (FMS), have shown limited predictive accuracy when used alone. At the same time, single-joint tests or anthropometric factors (e.g., BMI) are unlikely to capture the complex, multifactorial nature of non-contact lower-limb injury risk. A more comprehensive approach combines clinical joint stability tests, FMS measures, and intrinsic characteristics within a single analytical framework. Therefore, the primary aim of this study was to examine exploratory associations between Functional Movement Screen scores, lower-limb joint stability test findings, and subsequent non-contact lower-limb injury in collegiate athletes. A secondary objective was to evaluate the discriminatory performance of these candidate predictor sets using ridge-penalized logistic regression and cross-validated AUC.

## 2. Materials and Methods

### 2.1. Study Design and Participants

This study is a retrospective analysis of prospectively collected cohort data. Baseline joint stability and movement assessments were obtained during routine examinations before the competitive season, and injury outcomes were recorded longitudinally during the season. 381 male and female collegiate athletes from eleven sports were evaluated using BMI, the Functional Movement Screen (FMS), and clinical lower-limb joint tests before each sport’s 2015–2016 season (Table 1). Although the cohort is historical, the screening measures examined in this study remain clinically relevant because the Functional Movement Screen (FMS) and common lower-limb joint stability tests continue to be used in sports medicine and athletic screening contexts. Certified athletic trainers conducted the FMS and joint tests, with assistance from supervised athletic training students, during routine preseason medical evaluations. Injury history, injury occurrence, and the count of non-contact lower-limb injuries during the subsequent season were obtained from medical records maintained by the sports medicine staff. Before transfer, the sports medicine team deidentified and anonymized all data. Because the present analysis used existing, deidentified secondary data, it qualified for exempt status under federal regulations. The university’s Institutional Review Board granted exempt status (IRB# 23-032201).

All variables were screened for completeness and converted to appropriate numeric formats. Analyses were conducted using complete-case data for each model. Sex-stratified datasets were created for subgroup analyses, and derived variables were transformed as described below.

### 2.2. Outcome Measures

Because non-contact lower-limb injuries may occur as isolated events related to sport exposure, training load, or situational demands, classifying athletes solely by the occurrence of ≥1 injury may capture a heterogeneous outcome that includes both single-injury cases and athletes with higher seasonal injury burden. In contrast, sustaining ≥2 injuries during the season may better reflect an elevated injury burden and repeated susceptibility, particularly when evaluating intrinsic risk factors such as prior injury history, joint instability, BMI, and movement-screen performance. This threshold was not intended to define recurrent injury in the conventional sports medicine sense, because the available injury records did not establish whether subsequent injuries occurred at the same anatomical site, involved the same diagnosis, or followed recovery and return to sport. Rather, consistent with IOC injury and illness reporting recommendations, multiple injuries may result from different or similar events or etiologies, occur concurrently, or reflect a mixture of these patterns [18]. Therefore, the ≥2-injury threshold was selected as the primary outcome to identify athletes with a higher burden of non-contact lower-limb injury, while ≥1 injury was retained as a secondary outcome to assess whether findings were consistent under a broader injury definition.

### 2.3. Clinical Variables

**Figure 1:**
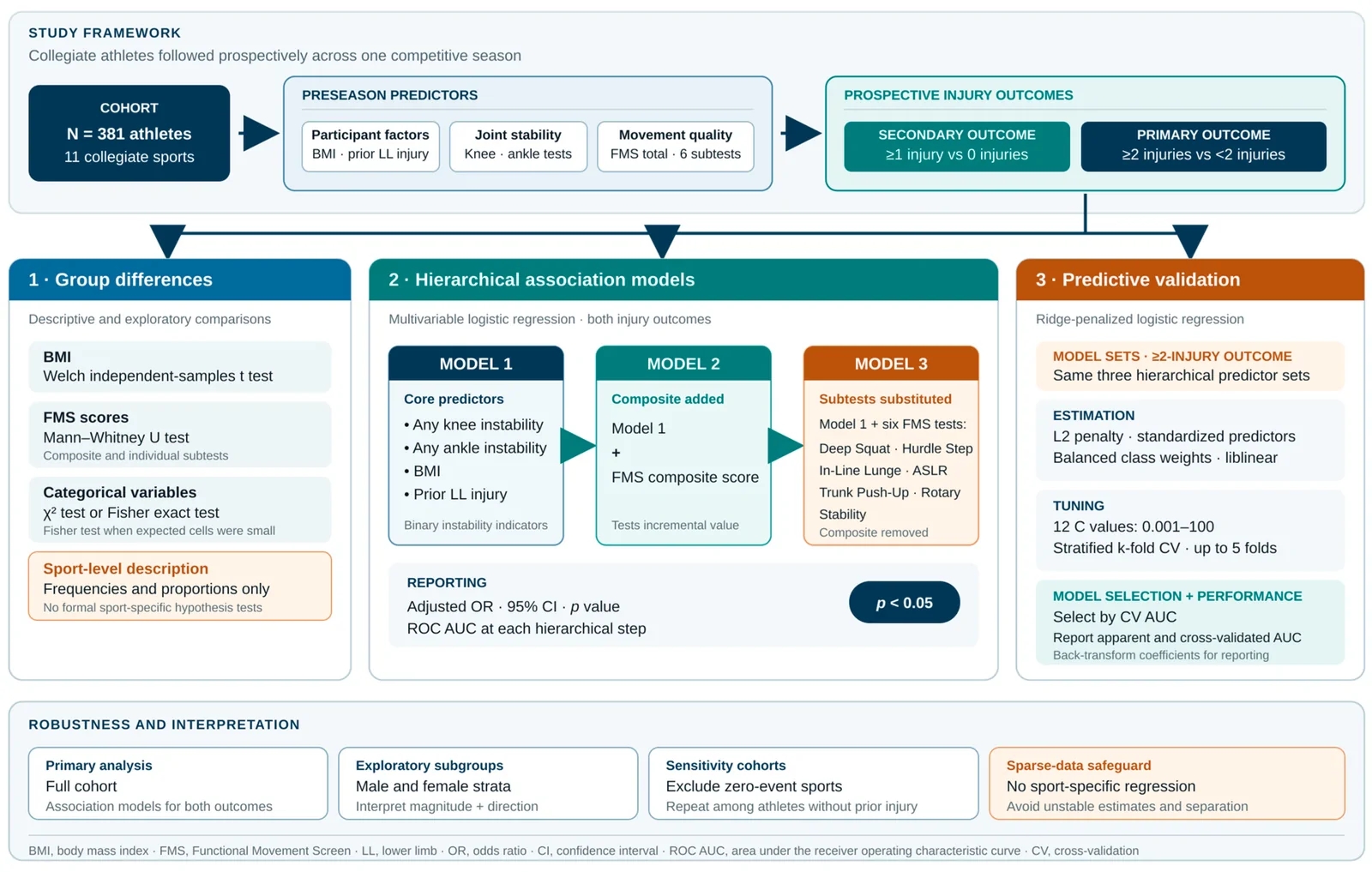
Analytic workflow.

Clinical variables included body mass index (BMI), history of lower-limb injury, Functional Movement Screen (FMS) measures (composite score and individual subtests, including Deep Squat, Hurdle Step, In-Line Lunge, Active Straight Leg Raise, Trunk Stability Push-Up, and Rotary Stability), and knee and ankle instability. The Shoulder Mobility test was removed to reduce noise in the models. Knee and ankle instability were defined using knee- and ankle-specific joint stability tests and were coded as dichotomous variables indicating the presence of at least one positive instability finding within each region (Table 1; Figure 1). Secondary analyses examined knee and ankle instability burden, defined as the total number of positive knee or ankle instability tests within each region (Figure 1). In accordance with the IOC recommendations, non-contact lower-limb injury was defined as an injury involving the hip/groin, thigh, knee, lower leg, ankle, or foot that was not caused by contact and for which an athlete sought medical care, as determined from clinician descriptions recorded in the electronic medical record [18]. Injury type, severity, diagnosis-specific recurrence, and time-loss classification were not assessed because they were outside the scope of this analysis.

### 2.4. Statistical Analyses

Descriptive statistics were computed for all variables. Continuous variables were summarized with means and standard deviations or medians and interquartile ranges, and categorical variables were summarized with frequencies and percentages. Normality was assessed using the Shapiro-Wilk test (*p* < 0.05). All analyses were conducted in Python (version 3.13.5; Wilmington, DE, USA) using the pandas, numpy, scikit-learn, and statsmodels libraries.

#### 2.4.1. Group Differences

Group differences were evaluated descriptively for both injury outcomes (≥1 injury vs. no injury and ≥2 injuries vs. <2 injuries). BMI was compared using Welch’s independent-samples t tests. The FMS composite score and FMS subtest scores were compared using Mann–Whitney U tests. Categorical variables were compared using chi-square tests of independence or Fisher’s exact tests when expected cell counts were small. These analyses were conducted for descriptive and exploratory purposes and were not adjusted for multiple comparisons. Sport-specific frequencies and injury occurrence rates were calculated to characterize injury distribution across teams. Formal sport-specific hypothesis testing was not performed because several sports contained small sample sizes and limited injury events, including zero-event strata for the ≥2 injury category.

#### 2.4.2. Hierarchical Logistic Regression Modeling

Hierarchical multivariable logistic regression was used to evaluate associations between preseason screening measures and subsequent injury outcomes and to assess the added value of the FMS composite score and FMS subtests. Models were constructed to reflect increasing levels of functional information. Model 1 included any knee and ankle instability, BMI, and prior lower-limb injury. Model 2 added the composite Functional Movement Screen (FMS) score. Because the FMS composite score is a summation of the FMS subtest scores, Model 3 replaced the FMS composite score with individual FMS subtests (Deep Squat, Hurdle Step, In-Line Lunge, Active Straight Leg Raise, Trunk Stability Push-Up, and Rotary Stability). Adjusted odds ratios (ORs), 95% confidence intervals (CIs), and *p*-values were reported. Odds ratios for the binary joint instability variables were interpreted as the change in injury odds associated with one or more positive instability findings. Statistical significance was defined as *p* < 0.05. Primary models were fit using the full sample. Sex-stratified analyses were conducted among male and female athletes to assess whether associations were consistent across sexes. Because sex stratification reduced the number of injury events available for estimation, sex-specific models were treated as exploratory subgroup analyses and interpreted primarily by effect magnitude and consistency rather than isolated statistical significance.

As a secondary analysis, ridge-penalized logistic regression was used to evaluate coefficient stability and cross-validated discrimination. For the primary ≥2-injury outcome, ridge-penalized logistic regression was fit using binary knee and ankle instability variables. Models were fit using L2 penalization with standardized candidate predictors, balanced class weights, and the liblinear solver. Hyperparameter tuning was performed using a grid search across 12 values of the inverse regularization parameter, C, ranging from 0.001 to 100.

Sport-level preliminary analyses showed substantial sparsity in the higher seasonal injury burden outcome variable (≥2 injuries), with several sports having few or no ≥2 injury events. Because of this sparsity, sport-specific logistic regression models were not fit due to the potential for unstable estimates and separation. Instead, injury rates were summarized descriptively by sport.

#### 2.4.3. Sensitivity Analyses

Several sensitivity analyses were conducted to assess the robustness of the model findings. First, analyses of the higher injury burden outcome were repeated after excluding sports with no ≥2 injury events. This approach was used to reduce the risk of unstable parameter estimates associated with sparse outcome distributions. A second analysis was repeated among athletes without a documented history of lower-limb injury at baseline. Because previous injury is one of the strongest known predictors of future injury, restricting analyses to previously uninjured athletes allowed evaluation of whether preseason instability and movement-screening measures remained associated with injury risk independent of prior injury history. The same hierarchical modeling framework used in the primary analyses was applied to the no-injury-history cohort.

### 2.5. Model Performance and Discrimination

Model discrimination was evaluated using receiver operating characteristic (ROC) curve analysis. The area under the ROC curve (AUC) was calculated for each hierarchical model to quantify the ability of preseason screening variables to distinguish between athletes who did and did not sustain injury. AUC values were computed for both injury outcomes (≥1 injury and ≥2 injuries), and model performance was compared across hierarchical steps to evaluate the incremental contribution of FMS measures. Ridge models were evaluated using stratified k-fold cross-validation, with the number of folds determined by the available number of outcome events and non-events, up to a maximum of five folds. Cross-validated AUC was used to select the optimal regularization parameter. Final model performance was summarized using apparent AUC and cross-validated AUC. Coefficients were backtransformed to the original variable scale for descriptive reporting. Therefore, odds ratios for binary variables represent the contrast between participants with and without the characteristic.

## 3. Results

### 3.1. Participant Characteristics and Injury Outcomes

A total of 381 collegiate athletes were included in the analysis, comprising both male (n = 246; 65%) and female (n = 135; 35%) participants across 11 sports (Table 2). The overall prevalence of at least one lower-limb non-contact injury (≥1 injury) was 21%, whereas 10.8% of athletes sustained two or more injuries (≥2 injuries). Injury outcomes varied by sex and sport. Injury burden was largely similar between males and females. Females had a slightly higher percentage of athletes with ≥1 injury (23.0%) than males (19.9%). Football accounted for the greatest absolute injury burden, whereas women’s volleyball and women’s soccer had the highest proportions of ≥2 injury cases. No ≥2 injury cases were observed in men’s basketball, women’s basketball, or women’s tennis.

### 3.2. Group Comparisons

For the primary group comparison (<2 injuries vs. ≥2 injuries), there were no significant differences in BMI, FMS composite score, FMS subtest scores, knee or ankle instability measures, and sport distribution did not differ significantly by injury status (Table 3). However, prior lower-limb injury history was significantly more prevalent among participants with ≥2 injuries than among those with <2 injuries (61% vs. 43%, *p* = 0.031). Similar findings were observed in the secondary injury-status comparison (no injury vs. ≥1 injury), in which prior lower-limb injury history was more prevalent among participants with ≥1 injury than among those with no injury (58% vs. 42%, *p* = 0.012; Table S1). In the secondary comparison, In-Line Lunge score distribution also differed significantly by injury status despite identical median scores between groups. Participants with ≥1 injury had a higher proportion of scores below 2 than those with no injury (12.5% vs. 7.6%) and a lower proportion of scores of 3 (5.0% vs. 11.6%; Mann–Whitney U *p* = 0.030; Table S2).

In the male-only stratum, sport distribution differed significantly between athletes with no injury and those with ≥1 injury (χ^2^(4) = 11.38, *p* = 0.023). Football players accounted for the largest share of injured male athletes (30/49; 61.2%), followed by men’s soccer players (12/49; 24.5%). However, when expressed as within-sport injury rates, ≥1 injury occurred in 26.1% of football players and 25.0% of men’s soccer players (Table S3). In the female-only stratum, BMI was significantly higher among those with ≥1 injury (26.13 ± 4.21) than among those with no injuries (24.26 ± 3.71; *p*-value = 0.031). In-Line Lunge score distribution also differed significantly by injury status, despite identical median scores between groups. Participants with ≥1 injury had a higher proportion of scores below 2 than those with no injury (12.9% vs. 5.8%) and a lower proportion of scores of 3 (6.5% vs. 23.1%; Mann–Whitney U *p* = 0.018; Tables S4 and S5).

### 3.3. Primary and Secondary Inferential Models

In unpenalized logistic regression models, prior lower-limb injury history was significantly associated with ≥2 injuries after adjustment for knee instability, ankle instability, and BMI in Model 1 (OR = 2.03, 95% CI [1.04, 3.96], *p* = 0.038) and after further adjustment for FMS composite score in Model 2 (OR = 2.00, 95% CI [1.02, 3.90], *p* = 0.044). Model 1 and Model 2 showed modest discrimination, with AUCs of 0.62 and 0.64, respectively. Results were similar in the secondary injury outcome group (≥1 injury). However, the In-Line Lunge test was significantly inversely associated with ≥1 injury after adjustment for knee instability, ankle instability, BMI, lower-limb injury history, and all other FMS subtests (OR = 0.51, 95% CI [0.28, 0.90], *p* = 0.021; AUC = 0.65; Table 4).

In the female-only stratum, using instability count variables, BMI was significantly associated with ≥1 injury after adjusting for knee instability count, ankle instability count, and lower-limb injury history in Model 1 (OR = 1.13, 95% CI [1.02, 1.26], *p* = 0.021, AUC = 0.70; Table S6). Lower-limb injury history was significantly associated with ≥2 injuries after adjusting for knee instability count, ankle instability count, and BMI in Model 1 (OR = 3.38, 95% CI [1.01, 11.35], *p* = 0.049, AUC = 0.66; Table S6). The Deep Squat was significantly inversely associated with ≥2 injuries after adjusting for knee instability count, ankle instability count, BMI, lower-limb injury history, and all other FMS subtests in Model 3 (OR = 0.29 [95% CI 0.09, 0.91], *p* = 0.035, AUC = 0.77; Table S6). Results were similar in female-only stratum models using any knee and ankle instability for BMI and lower-limb injury history in both injury outcome groups after adjusting for covariates in Model 1 (Table S7). However, in Model 1, lower-limb injury history was significantly associated with ≥1 injury (OR = 2.40 [95% CI 1.01, 5.70], *p* = 0.047, AUC = 0.70; Table S7). In Model 3, the In-Line Lunge was significantly inversely associated with ≥1 injury after covariate adjustment (OR = 0.37 [95% CI 0.14, 0.95], *p* = 0.039, AUC = 0.73; Table S7).

### 3.4. Sensitivity Models

In the no-history stratum, the In-Line Lunge was significantly inversely associated with ≥1 injury after adjusting for any knee or ankle instability, BMI, and all other FMS subtests (OR = 0.30 [95% CI 0.10, 0.90], *p* = 0.031, AUC = 0.70; Table S8). In the female-only, no injury history stratum, BMI was significantly associated with ≥1 injury after adjusting for covariates in Model 1 (OR = 1.25, 95% CI [1.05, 1.47], *p* = 0.01, AUC = 0.69; Table S9), Model 2 (OR = 1.23, 95% CI [1.03, 1.47], *p* = 0.024, AUC = 0.71; Table S9), and Model 3 (OR = 1.32, 95% CI [1.06, 1.66], *p* = 0.014, AUC = 0.80; Table S9). Results were similar using both the count and binary instability measures (Table S10). In the male-only, no injury history stratum, the In-Line Lunge was significantly inversely associated with ≥1 injury after adjusting for any knee and ankle instability, BMI, and all other FMS subtests in Model 3 (OR = 0.17, 95% CI [0.03, 0.87], *p* = 0.033, AUC = 0.75; Table S11). Any ankle instability was significantly associated with ≥2 injuries after adjusting for covariates in all three models (Model 1: OR = 4.22, 95% CI [1.03, 17.30], *p* = 0.045, AUC = 0.66, Table S11; Model 2: OR = 4.27, 95% CI [1.04, 17.54], *p* = 0.044, AUC = 0.66, Table S11; Model 3: OR = 4.46, 95% CI [1.03, 19.32], *p* = 0.046, AUC = 0.78, Table S11).

In the full sample after excluding sports with zero ≥2-injury events, prior lower-limb injury history remained significantly associated with ≥2 injuries across all binary-instability models (ORs = 2.07–2.16, *p* = 0.026–0.041; Table S12). The FMS composite score was also significant in Model 2 (OR = 0.83, *p* = 0.047), whereas knee and ankle instability were not significant. In the female-only sensitivity models, prior lower-limb injury history remained significant across Models 1–3 (ORs = 3.93–5.88, *p* = 0.025–0.030; Tables S13 and S14), and the FMS composite score was significant in Model 2 when both binary and count instability variables were used (OR = 0.64–0.65, *p* = 0.016–0.17; Tables S13 and S14). No knee or ankle instability variables were significant in the female-only models. Among male participants without prior lower-limb injury history, only ankle instability was significantly associated with ≥2 injuries after excluding zero-event sports, both when modeled as a count variable (ORs = 3.25–3.43, *p* = 0.033–0.047; Tables S15 and S16) and as a binary variable (ORs = 7.72–8.04, *p* = 0.010–0.013; Tables S14 and S15). BMI and FMS scores were not significant in these male no-history models. Overall, the sensitivity analyses suggest that prior injury history remained the most consistent association across the full and female-only samples. In contrast, ankle instability showed a stronger signal only among male athletes without a prior injury history.

### 3.5. Ridge-Penalized Logistic Regression

In ridge-penalized logistic regression models examining associations with ≥2 injuries, discrimination was modest and did not improve with adding FMS variables (Table 5). Model 1, which included any knee instability, any ankle instability, BMI, and prior lower-limb injury history, had an apparent AUC of 0.621 and a cross-validated AUC of 0.562. Adding the FMS composite score in Model 2 slightly increased the apparent AUC to 0.636 but did not improve cross-validated discrimination (CV AUC = 0.559). Model 3, which replaced the FMS composite score with individual FMS subtests, had the highest apparent AUC (0.649) but the lowest cross-validated AUC (0.535), suggesting limited generalizability. Cross-validated AUC values were slightly higher in models examining associations with ≥1 injury. Across all models, coefficients were strongly shrunk toward zero, and odds ratios were close to 1.00, indicating limited predictive contribution from instability, BMI, prior injury history, or FMS measures.

## 4. Discussion

This study aimed to determine whether combining FMS scores, BMI, lower-limb injury history, and joint-specific tests improved prediction of non-contact lower-limb injuries in collegiate athletes. The primary finding was that the combined preseason screening battery demonstrated limited discriminatory capacity, and adding the FMS composite score or individual FMS subtests did not improve cross-validated discrimination in the ridge models. Nevertheless, prior lower-limb injury history emerged as the most consistent individual clinical factor. Athletes with a prior lower-limb injury had approximately twice the odds of sustaining ≥2 non-contact lower-limb injuries during the subsequent season in the full sample and in sensitivity analyses excluding sports with no ≥2-injury cases, with stronger associations observed in the female-only models. Other associations were less consistent and should be interpreted cautiously because they were derived from overlapping subgroup and sensitivity analyses. Although not significantly associated with injury in the full sample, BMI was significantly higher among females, with and without prior injury history, who sustained at least one injury. Preseason knee and ankle special test findings did not show consistent associations with injury outcomes. However, ankle instability was significantly associated with higher injury burden among males without prior injury history. FMS findings were similarly inconsistent across models. The FMS composite score was significantly associated with ≥2 injuries in the full sample and in the female-only sample after excluding sports with no ≥2 injury cases, with higher FMS composite scores associated with lower odds of ≥2 injuries. The In-Line Lunge was inversely associated with the odds of sustaining at least one injury in the full sample and among athletes with no prior injury history, whereas the Deep Squat was inversely associated with ≥2 injuries in the female-only sample.

Previous research supports prior injury as a primary risk factor for future injury [19,20]. Two systematic reviews found that ACL injury was linked to multiple injuries and subsequent injuries at other anatomical locations, driven by decrements in strength, proprioception, tissue capacity, and altered neuromuscular control [19,20]. These findings support the notion that prior injury changes an athlete’s risk profile rather than representing an isolated event. In the current study, prior injury was associated with the broader ≥1-injury outcome and the higher-burden ≥2-injury outcome, suggesting that its influence was not limited to a single injury definition. However, injury history alone remains a risk marker rather than a sufficiently accurate predictive tool.

Several studies indicate that the FMS composite score has limited predictive value for injury. In a systematic review, Moran et al. reported a small association between the FMS composite score and subsequent injury, with conflicting evidence across study populations and analytic approaches [6]. Similarly, Dorrel found that a composite score of ≤15 was not a significant predictor of injury among Division II collegiate athletes and had limited ability to discriminate between athletes who would and would not sustain injury [21]. However, Mokha et al. reported that athletes with subtest asymmetries and low subtest scores were nearly 3 times more likely to sustain an injury than those without [22]. Therefore, summing the subtests into a single composite score may obscure important injury-risk information. These findings are broadly consistent with the present finding that the composite score was not independently associated with injury in the primary full-sample models and did not improve cross-validated AUC. However, the present study’s finding that the FMS composite score was associated with injury in the female-only sample is consistent with Chorba et al., who found that female collegiate athletes with composite scores ≤14 had approximately four times the risk of lower-limb injury, even among those without a prior history of injury [23]. In another prospective study of young volleyball players, Zarei et al. also identified an FMS score ≤14 as an injury risk factor [24]. The strength of associations between the FMS composite score and injury in the current study, however, was not as strong as those reported by [22] or [24]. This may reflect differences in population specificity, injury definitions, dichotomization of the FMS score, and sport exposure. Dichotomization can produce stronger associations when a sample-specific threshold is selected, while potentially removing information and reducing reproducibility.

The limited predictive contribution of the FMS composite score may also reflect a more fundamental measurement problem. The FMS composite score, a summation of the subtest scores, assumes that the seven movements are sufficiently interrelated to represent a common underlying construct, fundamental movement competence. However, this assumption has not been consistently supported. Kazman et al. reported low internal consistency among Marine officer candidates, concluding that the sum score was not supported as a unidimensional construct [25]. Li et al. similarly found low internal consistency and a multidimensional structure in elite athletes [26], while Kraus et al. reported weak relationships among several component scores and low internal consistency in their sample of recreational and semi-professional athletes [27]. Therefore, poor performance on one FMS movement may not be accompanied by poor performance on the others, and differences in subtest scores may offset one another when summed. The limited predictive value of the composite score in the present study may reflect both the multifactorial nature of injury and the psychometric limitations of combining heterogeneous movement tasks into a single score.

A higher In-Line Lunge score was associated with lower odds of sustaining at least one injury across the full sample, the female subgroup, the no-history subgroup, and the male no-history subgroup. The consistency of this finding across subsamples suggests that the In-Line Lunge may capture information not fully represented by the FMS composite score. The movement requires multiplanar lower-limb mobility, postural control, trunk stability, and control within a narrow base of support, all of which may be relevant to sport movements involving deceleration and changes in direction. However, this finding contrasts with Bardenett et al., in which injured Division I athletes had higher rather than lower In-Line Lunge scores [28]. In a related analysis of 167 Division I athletes, Warren et al. reported that athletes scoring 2 on the lunge were less likely to be injured than those scoring 3, suggesting that higher scores do not always correspond to lower injury risk in every population [12]. In the current study, the In-Line Lunge association was strongest for the ≥1-injury outcome and less certain for the primary ≥2-injury outcome. The identical group medians further demonstrate that the result reflected differences in the full ordinal score distribution rather than a large separation between groups. Replication in an independent cohort and evaluation of nonlinear or category-specific relationships are needed before using the In-Line Lunge to classify athletes as high or low risk.

Warranting similar caution, a higher Deep Squat score in the female-specific sample was associated with lower odds of two or more injuries in a fully adjusted female model, but the association was not consistent across instability definitions or in the full sample. The Deep Squat requires coordinated mobility and stability across the ankles, knees, hips, and trunk and may reflect a broader movement-control characteristic. However, the small number of female multiple-injury cases, the wide confidence interval, the number of subgroup comparisons, and the lack of cross-model consistency make a chance association plausible. These results warrant further evaluation of specific movement patterns and call into question the Deep Squat or In-Line Lunge as stand-alone screening tests.

BMI was associated with ≥1 injury among female athletes, including those without a prior lower-limb injury, but not in the overall sample. This aligns with studies suggesting that the relationship between body size and injury varies by sex and injury type [29,30]. Higher BMI has been linked to ACL injury risk in females, possibly because greater body mass increases joint loading when it is not matched by proportional strength or neuromuscular capacity [27]. Conversely, Warren et al. reported lower BMI among injured Division I athletes, indicating that BMI may operate differently across sports and injury mechanisms [12]. In another study, overweight athletes with a history of ankle sprains had 19 times the injury incidence of normal-weight athletes without prior injury, suggesting a strong interaction between BMI and injury history [31]. BMI also does not distinguish fat mass from lean mass, which is problematic in athletic populations. Therefore, the current study’s BMI findings should not necessarily be interpreted as a direct cause of injury. Instead, BMI may be used to assess its role as a mediator between specific exposures and injury outcomes.

The general lack of consistent associations between knee special test findings and injury conflicts with injury-specific studies of ligamentous laxity. Multiple studies found that greater knee laxity increased ACL injury risk across athletic and military samples [29,32]. These studies focused on a specific injury mechanism and used quantitative laxity measurements, whereas the present outcome combined multiple non-contact lower-limb injuries and defined knee instability using a heterogeneous set of clinical tests. Consequently, a positive joint test may be relevant to a particular ligament injury without an association with the broader occurrence of strains, tendinopathies, or injuries elsewhere in the lower extremity. However, Beynnon et al. reported that ankle instability was associated with two or more injuries among male athletes without prior lower-limb injury, and that increased talar tilt was linked to ankle ligament injury risk in males, whereas risk factors differed in females [33]. Similarly, a systematic review concluded that ankle-sprain risk factors differ between male and female athletes and emphasized previous ankle injury as an important risk factor in males [30]. These findings support a specific phenotypic susceptibility among some male athletes, even without a documented injury history. However, the associations between ankle instability and injury in the present study were based on a small number of injury events and had wide confidence intervals. Furthermore, the outcome was any non-contact lower-limb injury rather than an ankle-specific injury. These findings could indicate a sex-specific relationship, but they could also reflect sport composition or sparse-data bias. Future studies should evaluate ankle instability against anatomically matched ankle outcomes.

The ridge-penalized models underscore the distinction between identifying risk factors and accurately predicting which athlete will be injured. Although adding FMS variables initially appeared to improve the model’s performance (with the AUC rising from about 0.62 to 0.65–0.66), cross-validation showed no meaningful improvement, and the model that included all individual subtests performed the worst. This suggests that the added variables captured sample-specific variation rather than reliable injury information. Because the ridge coefficients shrank strongly toward zero, these preseason measures offer limited support as reliable predictors of injury. This aligns with Bahr’s argument that sports injuries are shaped by ever-changing interactions among an athlete’s characteristics, exposure, workload, opponents, and situational factors [34]. Thus, a single preseason screening is unlikely to reasonably predict injuries.

### 4.1. Clinical Considerations

Despite the low predictive value of FMS scores or joint tests, these measures may identify current pain, movement limitations, asymmetries, or laxity that warrant clinical attention. Moreover, their value for describing modifiable impairments or guiding further examination should be distinguished from their ability to predict future injury. The present results imply that preseason screening should not be used to clear, restrict, or label athletes based on a calculated probability. Greater emphasis should be placed on previous injury, the completion and quality of rehabilitation, current symptoms, sport-specific exposure, and ongoing monitoring of workload and neuromuscular status.

### 4.2. Limitations

This study has several limitations. First, while combining athletes from several sports increased generalizability across collegiate athletics, it introduced substantial heterogeneity in exposure and injury mechanisms. Moreover, competition and athlete exposure rates were unavailable, preventing calculation of exposure-adjusted injury incidence. Second, the ≥2-injury outcome identified higher injury burden but did not necessarily represent recurrence of the same injury or at the same anatomical site. Third, the number of multiple-injury cases was limited, particularly in sex-stratified and no-history analyses, resulting in uncertainty in the estimates. Fourth, multiple exploratory subgroup and sensitivity analyses were conducted without adjustment for multiple comparisons. Significant findings should be interpreted according to their consistency and effect estimates rather than their *p*-values alone. Fifth, the instability variables combined tests evaluating different joint structures and pathologies and did not account for severity, examiner reliability, symptoms, or ipsilateral correspondence with later injury. Sixth, several potentially important factors, including pre-existing injuries, exposure, training loads, strength, body composition, previous injury type and recency, rehabilitation status, playing position, and protective equipment, were unavailable. Finally, participant age was not captured during the data extraction process and could not be described or included as a covariate in the statistical models. Although the sample consisted of collegiate athletes and was expected to represent a relatively restricted age range, the absence of individual-level age data limits characterization of the cohort and prevents evaluation of age as a potential confounder or effect modifier. Future studies should focus on longitudinal, sport-specific monitoring to determine whether prescreening measures are associated with subsequent injury patterns, repeat injury occurrence, and higher seasonal injury burden.

## 5. Conclusions

In conclusion, a history of lower-limb injury was consistently associated with subsequent non-contact lower-limb injury. FMS and joint special-test measures provided little improvement in cross-validated discrimination. Some associations involving the In-Line Lunge, female BMI, female Deep Squat, and male ankle instability may indicate population-specific signals but require independent confirmation. These findings do not support using a single preseason screening battery to predict individual injury. Rather, they warrant a longitudinal, sport-specific approach that integrates detailed injury history, exposure, rehabilitation status, and repeated measures of physical and functional capacity.

## Figures and Tables

**Table 1:** Construction of Special Joint Test Instability Variables.

Variable Type	Variable Description	Variable Name	Original Variables	Coding/Calculation	Interpretation
Dichotomous joint test variable	Any positive special joint test	Any Knee Instability, Any Ankle Instability	Knee Valgus 0°, Knee Valgus 30°, Knee Varus 0°, Knee Varus 30°, Lachman test, Patellar Apprehension, Ankle Anterior Drawer, Inversion Talar Tilt, Eversion Talar Tilt assessed on the right and left legs	Coded as 1 if the participant had at least one positive test on either the right or left leg; coded as 0 if all tests were negative bilaterally	Indicates the presence or absence of any positive special joint test suggestive of knee joint instability or related pathology
Count joint test variable	Total number of positive special joint tests	Knee Instability Count, Ankle Instability Count		Calculated as the sum of all positive tests across the right and left legs	Represents the burden or extent of positive special joint findings, with higher values indicating a greater number of positive instability-related tests

**Table 2:** Distribution of Injury Outcomes by Sex and Sport.

Sport (N = 381)	N	≥1 Injury, n (%)	≥2 Injuries, n (%)
Males	246	49 (19.9%)	26 (10.6%)
Females	135	31 (23.0%)	15 (11.1%)
Baseball	45	3 (6.7%)	2 (4.4%)
Field hockey	23	7 (30.4%)	2 (8.7%)
Football	115	30 (26.1%)	17 (14.8%)
Men’s basketball	20	1 (5.0%)	0 (0.0%)
Men’s soccer	48	12 (25.0%)	6 (12.5%)
Men’s volleyball	18	3 (16.7%)	1 (5.6%)
Women’s basketball	15	4 (26.7%)	0 (0.0%)
Women’s lacrosse	26	3 (11.5%)	2 (7.7%)
Women’s soccer	34	9 (26.5%)	6 (17.6%)
Women’s tennis	13	2 (15.4%)	0 (0.0%)
Women’s volleyball	24	6 (25.0%)	5 (20.8%)

**Table 3:** Group Comparisons Between Participants With <2 Injuries and ≥2 Injuries.

Variable	Overall	<2 Injuries	≥2 Injuries	*p*-Value
BMI, kg/m^2^	26.45 ± 4.73	26.37 ± 4.75	27.04 ± 4.59	0.384
FMS composite score, 0–21	16.00 [14.00, 17.00]	16.00 [14.00, 17.00]	15.00 [13.00, 17.00]	0.085
Knee instability count: 0	312 (81.9%)	277 (81.5%)	35 (85.4%)	
Knee instability count: 1	37 (9.7%)	35 (10.3%)	2 (4.9%)	
Knee instability count: ≥2	32 (8.4%)	28 (8.2%)	4 (9.8%)	
Ankle instability count: 0	302 (79.3%)	271 (79.7%)	31 (75.6%)	
Ankle instability count: 1	58 (15.2%)	50 (14.7%)	8 (19.5%)	
Ankle instability count: ≥2	21 (5.5%)	19 (5.6%)	2 (4.9%)	
Deep Squat, 0–3	2.00 [2.00, 2.00]	2.00 [2.00, 2.00]	2.00 [2.00, 2.00]	0.511
Hurdle Step, 0–3	2.00 [2.00, 2.00]	2.00 [2.00, 2.00]	2.00 [2.00, 2.00]	0.673
In-Line Lunge, 0–3	2.00 [2.00, 2.00]	2.00 [2.00, 2.00]	2.00 [2.00, 2.00]	0.142
Active Straight Leg Raise, 0–3	3.00 [2.00, 3.00]	3.00 [2.00, 3.00]	2.00 [2.00, 3.00]	0.104
Trunk Stability Push-Up, 0–3	3.00 [2.00, 3.00]	3.00 [2.00, 3.00]	3.00 [2.00, 3.00]	0.879
Rotary Stability, 0–3	2.00 [2.00, 2.00]	2.00 [2.00, 2.00]	2.00 [2.00, 2.00]	0.836
Sex: Female, n (%)	135 (35.4%)	120 (35.3%)	15 (36.6%)	0.864
Sex: Male, n (%)	246 (64.6%)	220 (64.7%)	26 (63.4%)	
Prior lower-limb injury history: No, n (%)	210 (55.1%)	194 (57.1%)	16 (39.0%)	**0.031**
Prior lower-limb injury history: Yes, n (%)	171 (44.9%)	146 (42.9%)	25 (61.0%)	
Any knee instability: No, n (%)	312 (81.9%)	277 (81.5%)	35 (85.4%)	0.670
Any knee instability: Yes, n (%)	69 (18.1%)	63 (18.5%)	6 (14.6%)	
Any ankle instability: No, n (%)	302 (79.3%)	271 (79.7%)	31 (75.6%)	0.543
Any ankle instability: Yes, n (%)	79 (20.7%)	69 (20.3%)	10 (24.4%)	
Sport: Baseball, n (%)	45 (11.8%)	43 (12.6%)	2 (4.9%)	0.139
Sport: Field hockey, n (%)	23 (6.0%)	21 (6.2%)	2 (4.9%)	
Sport: Football, n (%)	115 (30.2%)	98 (28.8%)	17 (41.5%)	
Sport: Men’s basketball, n (%)	20 (5.2%)	20 (5.9%)	0 (0.0%)	
Sport: Men’s soccer, n (%)	48 (12.6%)	42 (12.4%)	6 (14.6%)	
Sport: Men’s volleyball, n (%)	18 (4.7%)	17 (5.0%)	1 (2.4%)	
Sport: Women’s basketball, n (%)	15 (3.9%)	15 (4.4%)	0 (0.0%)	
Sport: Women’s lacrosse, n (%)	26 (6.8%)	24 (7.1%)	2 (4.9%)	
Sport: Women’s soccer, n (%)	34 (8.9%)	28 (8.2%)	6 (14.6%)	
Sport: Women’s tennis, n (%)	13 (3.4%)	13 (3.8%)	0 (0.0%)	
Sport: Women’s volleyball, n (%)	24 (6.3%)	19 (5.6%)	5 (12.2%)	

Values are presented as mean ± SD, median [IQR], or n (%). BMI = body mass index; FMS = Functional Movement Screen. BMI was compared using Welch’s *t*-test. FMS composite and FMS component scores were compared using Mann-Whitney U tests. Binary categorical variables were compared using Fisher exact tests. Sport was compared using a chi-square test of the overall sport distribution between injury groups. Bolded *p*-values indicate *p* < 0.05. Because some expected cell counts were low, the sport-distribution *p*-value should be interpreted cautiously.

**Table 4:** Adjusted Logistic Regression Models for Lower-Limb Injury Outcomes in the Full Sample (N = 381).

Outcome	Model	Variable	OR	95% CI	*p*-Value	AUC
≥1 injury	1	Knee instability (any)	0.69	0.33, 1.41	0.308	0.62
		Ankle instability (any)	1.30	0.71, 2.39	0.401	
		BMI	1.04	0.99, 1.10	0.123	
		LL injury history	1.86	1.12, 3.08	**0.016**	
	2	Knee instability (any)	0.67	0.32, 1.38	0.278	0.63
		Ankle instability (any)	1.29	0.70, 2.38	0.412	
		BMI	1.03	0.98, 1.09	0.254	
		LL injury history	1.84	1.11, 3.06	**0.018**	
		FMS composite score	0.92	0.80, 1.05	0.207	
	3	Knee instability (any)	0.63	0.30, 1.32	0.218	0.65
		Ankle instability (any)	1.22	0.65, 2.27	0.535	
		BMI	1.03	0.97, 1.08	0.381	
		LL injury history	1.72	1.03, 2.88	**0.038**	
		Deep Squat	1.10	0.68, 1.79	0.692	
		Hurdle Step	0.96	0.59, 1.57	0.872	
		In-Line Lunge	0.51	0.28, 0.90	**0.021**	
		Active Straight Leg Raise	0.85	0.55, 1.33	0.481	
		Trunk Stability Push-Up	1.18	0.77, 1.81	0.446	
		Rotary Stability	1.14	0.57, 2.27	0.706	
≥2 injuries	1	Knee instability (any)	0.75	0.29, 1.92	0.554	0.62
		Ankle instability (any)	1.25	0.57, 2.74	0.571	
		BMI	1.03	0.96, 1.10	0.473	
		LL injury history	2.03	1.04, 3.96	**0.038**	
	2	Knee instability (any)	0.72	0.28, 1.86	0.499	0.64
		Ankle instability (any)	1.25	0.57, 2.75	0.579	
		BMI	1.01	0.94, 1.08	0.803	
		LL injury history	2.00	1.02, 3.90	**0.044**	
		FMS composite score	0.86	0.72, 1.02	0.089	
	3	Knee instability (any)	0.67	0.26, 1.78	0.426	0.66
		Anke instability (any)	1.21	0.54, 2.70	0.637	
		BMI	1.01	0.93, 1.08	0.859	
		LL injury history	1.88	0.95, 3.71	0.070	
		Deep Squat	0.76	0.42, 1.39	0.375	
		Hurdle Step	1.22	0.64, 2.32	0.551	
		In-Line Lunge	0.55	0.27, 1.09	0.087	
		Active Straight Leg Raise	0.66	0.37, 1.16	0.146	
		Trunk Stability Push-Up	1.19	0.68, 2.08	0.545	
		Rotary Stability	0.95	0.41, 2.17	0.899	

Model 1 included knee instability status, ankle instability status, BMI, and prior lower-limb injury history. Model 2 added the FMS composite score. Model 3 replaced the FMS composite score with individual FMS component scores. OR = odds ratio; CI = confidence interval; AUC = area under the receiver operating characteristic curve; BMI = body mass index; LL = lower limb; FMS = Functional Movement Screen. Bolded *p*-values indicate *p* < 0.05.

**Table 5:** Ridge-Penalized Logistic Regression Models for Injury Outcomes: Full Sample (N = 381).

Outcome	Model	Variable	Coefficient	OR per SD	Unstandardized OR	Apparent AUC	CV AUC	Best C	N	Events	EPV
≥1 injury	Model 1: Instability + BMI + history	Intercept	−0.010			0.622	0.591	0.008	381	80	20.00
		Any knee instability	−0.064	0.938	0.85						
		Any ankle instability	0.045	1.046	1.12						
		BMI	0.091	1.095	1.02						
		Prior lower-limb injury history	0.138	1.148	1.32						
	Model 2: Model 1 + Total FMS	Intercept	−0.000			0.626	0.599	0.001	381	80	16.00
		Any knee instability	−0.013	0.988	0.97						
		Any ankle instability	0.008	1.008	1.02						
		BMI	0.018	1.019	1.004						
		Prior lower-limb injury history	0.028	1.028	1.06						
		FMS composite score	−0.018	0.982	0.99						
	Model 3: Model 1 + FMS subtests	Intercept	−0.039			0.649	0.569	0.023	381	80	8.00
		Any knee instability	−0.121	0.886	0.73						
		Any ankle instability	0.064	1.066	1.17						
		BMI	0.102	1.108	1.02						
		Prior lower-limb injury history	0.202	1.224	1.50						
		Deep Squat	0.031	1.032	1.06						
		Hurdle Step	−0.027	0.973	0.95						
		In-Line Lunge	−0.211	0.809	0.64						
		Active Straight Leg Raise	−0.072	0.930	0.88						
		Trunk Stability Push-Up	0.063	1.065	1.11						
		Rotary Stability	0.040	1.041	1.12						
≥2 injuries	Model 1: Instability + BMI + history	Intercept	−0.001	0.999		0.621	0.562	0.001	381	41	10.25
		Any knee instability	−0.009	0.991	0.98						
		Any ankle instability	0.009	1.009	1.02						
		BMI	0.012	1.012	1.00						
		Prior lower-limb injury history	0.031	1.032	1.06						
	Model 2: Model 1 + Total FMS	Intercept	−0.001	0.999		0.636	0.559	0.001	381	41	8.20
		Any knee instability	−0.009	0.991	0.98						
		Any ankle instability	0.009	1.009	1.02						
		BMI	0.012	1.012	1.002						
		Prior lower-limb injury history	0.031	1.032	1.06						
		FMS composite score	−0.027	0.974	0.99						
	Model 3: Model 1 + FMS subtests	Intercept	−0.001	0.999		0.649	0.535	0.001	381	41	4.10
		Any knee instability	−0.010	0.991	0.98						
		Any ankle instability	0.009	1.009	1.02						
		BMI	0.011	1.011	1.002						
		Prior lower-limb injury history	0.031	1.031	1.06						
		Deep Squat	−0.017	0.983	0.97						
		Hurdle Step	−0.004	0.996	0.99						
		In-Line Lunge	−0.029	0.972	0.94						
		Active Straight Leg Raise	−0.023	0.977	0.96						
		Trunk Stability Push-Up	0.004	1.004	1.007						
		Rotary Stability	−0.005	0.996	0.99						

Values are ridge-penalized coefficients and odds ratios after variable standardization. Apparent AUC reflects model performance in the analytic sample, whereas CV AUC reflects the best cross-validated AUC during tuning. AUC = area under the receiver operating characteristic curve; CV = cross-validation; OR = odds ratio; FMS = Functional Movement Screen; BMI = body mass index; EPV = events per variable.

## Data Availability

The raw data supporting the conclusions of this article will be made available by the authors on request.

## Supplementary Materials

The following supporting information can be downloaded at: https://www.mdpi.com/article/10.3390/jcm15166466/s1, Table S1: Group Comparisons Between Participants with No Injury and ≥1 Injury; Table S2: Distribution of Functional Movement Screen Component Scores by Injury Group; Table S3: Male-Only Group Differences by ≥1 Lower-Limb Injury Status; Table S4: Female-Only Group Differences by ≥1 Lower-Limb Injury Status; Table S5: Female-Only FMS Score Distribution by No Injury vs ≥1 Lower-Limb Injury Status; Table S6: Adjusted Logistic Regression Models for Lower-Limb Injury Outcomes Among Female Participants; Table S7: Adjusted Logistic Regression Models for Lower-Limb Injury Outcomes Among Female Participants using Binary Instability Variables (N = 135); Table S8: Adjusted Logistic Regression Models for Lower-Limb Injury Outcomes Among Participants Without Prior Lower-Limb Injury History Using Binary Instability Variables (N = 210); Table S9: Adjusted Logistic Regression Models for Lower-Limb Injury Outcomes Among Female Participants Without Prior Lower-Limb Injury History; Table S10: Adjusted Logistic Regression Models for Lower-Limb Injury Outcomes Among Female Participants Without Prior Lower-Limb Injury History Using Binary Instability Variables (N = 69); Table S11: Adjusted Logistic Regression Models for Lower-Limb Injury Outcomes Among Male Participants Without Prior Lower-Limb Injury History Using Binary Instability Variables (N = 141); Table S12: Sensitivity Analysis: Adjusted Logistic Regression Models for ≥2 Lower-Limb Injuries in the Full Sample After Excluding Sports with Zero Events Using Binary Instability Variables; Table S13: Sensitivity Analysis: Adjusted Logistic Regression Models for ≥2 Lower-Limb Injuries Among Female Participants After Excluding Zero-Event Sports; Table S14: Sensitivity Analysis: Adjusted Logistic Regression Models for ≥2 Lower-Limb Injuries Among Female Participants After Excluding Sports with Zero Events Using Binary Instability Variables; Table S15: Sensitivity Analysis: Adjusted Logistic Regression Models for ≥2 Lower-Limb Injuries Among Male Participants Without Prior Lower-Limb Injury History After Excluding Zero-Event Sports; Table S16: Sensitivity Analysis: Adjusted Logistic Regression Models for ≥2 Lower-Limb Injuries Among Male Participants Without Prior Lower-Limb Injury History After Excluding Sports with Zero Events Using Binary Instability Variables.
