# Supplementary material for: Associations of Functional Movement Screen Scores and Joint Stability Tests with Non-Contact Lower-Limb Injury Burden in Collegiate Athletes
Source: J Clin Med. 2026 Aug 21;15(16):6466. doi: 10.3390/jcm15166466 (PMC13514592; doi:10.3390/jcm15166466)
Supplement: Supplementary file 1 [file jcm-15-06466-s001.zip › jcm-4498113-supplementary.pdf]

Table S1. *Group Comparisons Between Participants with No Injury and ≥1 Injury*

Variable	Overall	No injury	≥1 injury	p-value
BMI, kg/m²	26.45 ± 4.73	26.23 ± 4.66	27.27 ± 4.95	.092
FMS composite score, 0–21	16.00 [14.00, 17.00]	16.00 [14.00, 17.00]	15.00 [14.00, 16.25]	.100
Knee instability count: 0	312 (81.9%)	243 (80.7%)	69 (86.2%)	—
Knee instability count: 1	37 (9.7%)	32 (10.6%)	5 (6.2%)	
Knee instability count: ≥2	32 (8.4%)	26 (8.6%)	6 (7.5%)	
Ankle instability count: 0	302 (79.3%)	241 (80.1%)	61 (76.2%)	—
Ankle instability count: 1	58 (15.2%)	45 (15.0%)	13 (16.2%)	
Ankle instability count: ≥2	21 (5.5%)	15 (5.0%)	6 (7.5%)	
Deep Squat, 0–3	2.00 [2.00, 2.00]	2.00 [2.00, 2.00]	2.00 [2.00, 2.00]	.722
Hurdle Step, 0–3	2.00 [2.00, 2.00]	2.00 [2.00, 2.00]	2.00 [2.00, 2.00]	.261
In-Line Lunge, 0–3	2.00 [2.00, 2.00]	2.00 [2.00, 2.00]	2.00 [2.00, 2.00]	**.030**
Active Straight Leg Raise, 0–3	3.00 [2.00, 3.00]	3.00 [2.00, 3.00]	3.00 [2.00, 3.00]	.248
Trunk Stability Push-Up, 0–3	3.00 [2.00, 3.00]	3.00 [2.00, 3.00]	3.00 [2.00, 3.00]	.562
Rotary Stability, 0–3	2.00 [2.00, 2.00]	2.00 [2.00, 2.00]	2.00 [2.00, 2.00]	.714
Sex: Female, n (%)	135 (35.4%)	104 (34.6%)	31 (38.8%)	.512
Sex: Male, n (%)	246 (64.6%)	197 (65.4%)	49 (61.3%)	
Prior lower-limb injury history: No, n (%)	210 (55.1%)	176 (58.5%)	34 (42.5%)	**.012**
Prior lower-limb injury history: Yes, n (%)	171 (44.9%)	125 (41.5%)	46 (57.5%)	
Any knee instability: No, n (%)	312 (81.9%)	243 (80.7%)	69 (86.2%)	.327
Any knee instability: Yes, n (%)	69 (18.1%)	58 (19.3%)	11 (13.8%)	
Any ankle instability: No, n (%)	302 (79.3%)	241 (80.1%)	61 (76.2%)	.442
Any ankle instability: Yes, n (%)	79 (20.7%)	60 (19.9%)	19 (23.8%)	
Baseball	45 (11.8%)	42 (14.0%)	3 (3.8%)	.127

Variable	Overall	No injury	≥1 injury	p-value
Field hockey	23 (6.0%)	16 (5.3%)	7 (8.8%)	
Football	115 (30.2%)	85 (28.2%)	30 (37.5%)	
Men’s basketball	20 (5.2%)	19 (6.3%)	1 (1.2%)	
Men’s soccer	48 (12.6%)	36 (12.0%)	12 (15.0%)	
Men’s volleyball	18 (4.7%)	15 (5.0%)	3 (3.8%)	
Women’s basketball	15 (3.9%)	11 (3.7%)	4 (5.0%)	
Women’s lacrosse	26 (6.8%)	23 (7.6%)	3 (3.8%)	
Women’s soccer	34 (8.9%)	25 (8.3%)	9 (11.2%)	
Women’s tennis	13 (3.4%)	11 (3.7%)	2 (2.5%)	
Women’s volleyball	24 (6.3%)	18 (6.0%)	6 (7.5%)	

Values are presented as mean ± SD, median [IQR], or n (%). BMI = body mass index; FMS = Functional Movement Screen. BMI was compared using Welch’s t-test. FMS composite and FMS component scores were compared using Mann-Whitney U tests. Binary categorical variables were compared using Fisher exact tests. Sport was compared using a chi-square test of the overall sport distribution between injury groups. Because some expected cell counts were low, the sport-distribution p-value should be interpreted cautiously. Bolded p-values are significant at $p < .05$.

Table S2. *Distribution of Functional Movement Screen Component Scores by Injury Group*

Comparison	FMS component	Score	Overall, n (%)	Reference group, n (%)	Injury group, n (%)
No injury vs ≥1 injury	Deep Squat	0	6 (1.6%)	3 (1.0%)	3 (3.8%)
		1	28 (7.3%)	24 (8.0%)	4 (5.0%)
		2	287 (75.3%)	228 (75.7%)	59 (73.8%)
		3	60 (15.7%)	46 (15.3%)	14 (17.5%)
	Hurdle Step	0	0 (0.0%)	0 (0.0%)	0 (0.0%)
		1	33 (8.7%)	23 (7.6%)	10 (12.5%)
		2	257 (67.5%)	204 (67.8%)	53 (66.2%)

Comparison	FMS component	Score	Overall, n (%)	Reference group, n (%)	Injury group, n (%)
		3	91 (23.9%)	74 (24.6%)	17 (21.2%)
	In-Line Lunge	0	5 (1.3%)	1 (0.3%)	4 (5.0%)
		1	28 (7.3%)	22 (7.3%)	6 (7.5%)
		2	309 (81.1%)	243 (80.7%)	66 (82.5%)
		3	39 (10.2%)	35 (11.6%)	4 (5.0%)
	Active Straight Leg Raise	0	0 (0.0%)	0 (0.0%)	0 (0.0%)
		1	19 (5.0%)	14 (4.7%)	5 (6.2%)
		2	141 (37.0%)	108 (35.9%)	33 (41.2%)
		3	221 (58.0%)	179 (59.5%)	42 (52.5%)
	Trunk Stability Push-Up	0	1 (0.3%)	1 (0.3%)	0 (0.0%)
		1	22 (5.8%)	17 (5.6%)	5 (6.2%)
		2	156 (40.9%)	126 (41.9%)	30 (37.5%)
		3	202 (53.0%)	157 (52.2%)	45 (56.2%)
	Rotary Stability	0	6 (1.6%)	4 (1.3%)	2 (2.5%)
		1	18 (4.7%)	15 (5.0%)	3 (3.8%)
		2	347 (91.1%)	275 (91.4%)	72 (90.0%)
		3	10 (2.6%)	7 (2.3%)	3 (3.8%)
<2 injuries vs ≥2 injuries	Deep Squat	0	6 (1.6%)	3 (0.9%)	3 (7.3%)
		1	28 (7.3%)	26 (7.6%)	2 (4.9%)
		2	287 (75.3%)	257 (75.6%)	30 (73.2%)
		3	60 (15.7%)	54 (15.9%)	6 (14.6%)
	Hurdle Step	0	0 (0.0%)	0 (0.0%)	0 (0.0%)
		1	33 (8.7%)	30 (8.8%)	3 (7.3%)
		2	257 (67.5%)	227 (66.8%)	30 (73.2%)
		3	91 (23.9%)	83 (24.4%)	8 (19.5%)
	In-Line Lunge	0	5 (1.3%)	2 (0.6%)	3 (7.3%)
		1	28 (7.3%)	26 (7.6%)	2 (4.9%)

Comparison	FMS component	Score	Overall, n (%)	Reference group, n (%)	Injury group, n (%)
		2	309 (81.1%)	275 (80.9%)	34 (82.9%)
		3	39 (10.2%)	37 (10.9%)	2 (4.9%)
	Active Straight Leg Raise	0	0 (0.0%)	0 (0.0%)	0 (0.0%)
		1	19 (5.0%)	16 (4.7%)	3 (7.3%)
		2	141 (37.0%)	122 (35.9%)	19 (46.3%)
		3	221 (58.0%)	202 (59.4%)	19 (46.3%)
	Trunk Stability Push-Up	0	1 (0.3%)	1 (0.3%)	0 (0.0%)
		1	22 (5.8%)	20 (5.9%)	2 (4.9%)
		2	156 (40.9%)	139 (40.9%)	17 (41.5%)
		3	202 (53.0%)	180 (52.9%)	22 (53.7%)
	Rotary Stability	0	6 (1.6%)	4 (1.2%)	2 (4.9%)
		1	18 (4.7%)	16 (4.7%)	2 (4.9%)
		2	347 (91.1%)	313 (92.1%)	34 (82.9%)
		3	10 (2.6%)	7 (2.1%)	3 (7.3%)

The table displays the distribution of Functional Movement Screen component scores across two injury-status comparisons: no injury versus ≥1 injury and <2 injuries versus ≥2 injuries. Values are presented as n (%). Percentages are calculated within the overall analytic sample and within each injury-status group for the respective comparison. FMS = Functional Movement Screen. Component scores range from 0 to 3, with higher scores indicating better movement performance. For the no injury versus ≥1 injury comparison, the no-injury group included 301 participants and the ≥1-injury group included 80 participants. For the <2 injuries versus ≥2 injuries comparison, the <2-injury group included 340 participants and the ≥2-injury group included 41 participants.

Table S3. *Male-Only Group Differences by ≥1 Lower-Limb Injury Status*

Variable	Overall	No injury	≥1 injury	p-value
BMI, kg/m^2	27.41 ± 4.88	27.26 ± 4.78	27.99 ± 5.28	.381
FMS composite score, 0–21	15.00 [14.00, 16.00]	15.00 [14.00, 16.00]	15.00 [14.00, 16.00]	.267
Knee instability count: 0	210 (85.4%)	165 (83.8%)	45 (91.8%)	—
Knee instability count: 1	22 (8.9%)	19 (9.6%)	3 (6.1%)	

Variable	Overall	No injury	≥1 injury	p-value
Knee instability count: ≥2	14 (5.7%)	13 (6.6%)	1 (2.0%)	
Ankle instability count: 0	200 (81.3%)	162 (82.2%)	38 (77.6%)	—
Ankle instability count: 1	33 (13.4%)	25 (12.7%)	8 (16.3%)	
Ankle instability count: ≥2	13 (5.3%)	10 (5.1%)	3 (6.1%)	
Deep Squat, 0–3	2.00 [2.00, 2.00]	2.00 [2.00, 2.00]	2.00 [2.00, 2.00]	.436
Hurdle Step, 0–3	2.00 [2.00, 2.00]	2.00 [2.00, 2.00]	2.00 [2.00, 2.00]	.673
In-Line Lunge, 0–3	2.00 [2.00, 2.00]	2.00 [2.00, 2.00]	2.00 [2.00, 2.00]	.389
Active Straight Leg Raise, 0–3	2.00 [2.00, 3.00]	3.00 [2.00, 3.00]	2.00 [2.00, 3.00]	.147
Trunk Stability Push-Up, 0–3	3.00 [2.00, 3.00]	3.00 [2.00, 3.00]	3.00 [2.00, 3.00]	.482
Rotary Stability, 0–3	2.00 [2.00, 2.00]	2.00 [2.00, 2.00]	2.00 [2.00, 2.00]	.969
Prior lower-limb injury history: No, n (%)	141 (57.3%)	118 (59.9%)	23 (46.9%)	.109
Prior lower-limb injury history: Yes, n (%)	105 (42.7%)	79 (40.1%)	26 (53.1%)	
Any knee instability: No, n (%)	210 (85.4%)	165 (83.8%)	45 (91.8%)	.181
Any knee instability: Yes, n (%)	36 (14.6%)	32 (16.2%)	4 (8.2%)	
Any ankle instability: No, n (%)	200 (81.3%)	162 (82.2%)	38 (77.6%)	.539
Any ankle instability: Yes, n (%)	46 (18.7%)	35 (17.8%)	11 (22.4%)	
Sport: Baseball, n (%)	45 (18.3%)	42 (21.3%)	3 (6.1%)	**.023**
Sport: Football, n (%)	115 (46.7%)	85 (43.1%)	30 (61.2%)	
Sport: Men's basketball, n (%)	20 (8.1%)	19 (9.6%)	1 (2.0%)	
Sport: Men's soccer, n (%)	48 (19.5%)	36 (18.3%)	12 (24.5%)	
Sport: Men's volleyball, n (%)	18 (7.3%)	15 (7.6%)	3 (6.1%)	

The table displays male-only comparisons between participants with no injury and ≥1 injury. The analysis was restricted to male participants (N = 246), including 197 participants with no injury and 49 with ≥1 injury. Values are presented as mean ± SD, median [Q1, Q3], or n (%). Percentages are column percentages. BMI was compared using Welch's independent-samples t test. Total FMS and FMS component scores were compared using Mann–Whitney U tests. Binary categorical variables were compared using Fisher's exact test. Sport distribution was compared using chi-square analysis, although expected cell counts were low. BMI = body mass index; FMS = Functional Movement Screen; SD = standard deviation; Q1 = first quartile; Q3 = third quartile. Bolded p-values are significant at $p < .05$.

Table S4. *Female-Only Group Differences by ≥1 Lower-Limb Injury Status*

Variable	Overall	No injury	≥1 injury	p-value
BMI, kg/m²	24.69 ± 3.89	24.26 ± 3.71	26.13 ± 4.21	**.031**
FMS composite score, 0–21	16.00 [15.00, 17.00]	16.00 [15.00, 17.00]	15.00 [14.50, 16.50]	.139
Knee instability count: 0	102 (75.6%)	78 (75.0%)	24 (77.4%)	—
Knee instability count: 1	15 (11.1%)	13 (12.5%)	2 (6.5%)	
Knee instability count: ≥2	18 (13.3%)	13 (12.5%)	5 (16.1%)	
Ankle instability count: 0	102 (75.6%)	79 (76.0%)	23 (74.2%)	—
Ankle instability count: 1	25 (18.5%)	20 (19.2%)	5 (16.1%)	
Ankle instability count: ≥2	8 (5.9%)	5 (4.8%)	3 (9.7%)	
Deep Squat, 0–3	2.00 [2.00, 2.00]	2.00 [2.00, 2.00]	2.00 [2.00, 2.00]	.596
Hurdle Step, 0–3	2.00 [2.00, 3.00]	2.00 [2.00, 3.00]	2.00 [2.00, 2.00]	.147
In-Line Lunge, 0–3	2.00 [2.00, 2.00]	2.00 [2.00, 2.00]	2.00 [2.00, 2.00]	**.018**
Active Straight Leg Raise, 0–3	3.00 [2.00, 3.00]	3.00 [2.00, 3.00]	3.00 [2.00, 3.00]	.761
Trunk Stability Push-Up, 0–3	2.00 [2.00, 3.00]	2.00 [2.00, 3.00]	2.00 [2.00, 3.00]	.855
Rotary Stability, 0–3	2.00 [2.00, 2.00]	2.00 [2.00, 2.00]	2.00 [2.00, 2.00]	.542
Prior lower-limb injury history: No, n (%)	69 (51.1%)	58 (55.8%)	11 (35.5%)	.065
Prior lower-limb injury history: Yes, n (%)	66 (48.9%)	46 (44.2%)	20 (64.5%)	
Any knee instability: No, n (%)	102 (75.6%)	78 (75.0%)	24 (77.4%)	1.000
Any knee instability: Yes, n (%)	33 (24.4%)	26 (25.0%)	7 (22.6%)	
Any ankle instability: No, n (%)	102 (75.6%)	79 (76.0%)	23 (74.2%)	.816
Any ankle instability: Yes, n (%)	33 (24.4%)	25 (24.0%)	8 (25.8%)	
Sport: Field hockey, n (%)	23 (17.0%)	16 (15.4%)	7 (22.6%)	.627
Sport: Women's basketball, n (%)	15 (11.1%)	11 (10.6%)	4 (12.9%)	
Sport: Women's lacrosse, n (%)	26 (19.3%)	23 (22.1%)	3 (9.7%)	
Sport: Women's soccer, n (%)	34 (25.2%)	25 (24.0%)	9 (29.0%)	
Sport: Women's tennis, n (%)	13 (9.6%)	11 (10.6%)	2 (6.5%)	
Sport: Women's volleyball, n (%)	24 (17.8%)	18 (17.3%)	6 (19.4%)	

The table displays female-only comparisons between participants with no injury and ≥1 injury. The analysis was restricted to female participants (N = 135), including 104 participants with no injury and 31 with ≥1 injury. Values are presented as mean ± SD, median [Q1, Q3], or n (%). Percentages are column percentages. BMI was compared using Welch's independent-samples t test. Total FMS and FMS component scores were compared using Mann–Whitney U tests. Binary categorical variables were compared using Fisher's exact test. Sport distribution was compared using chi-square analysis, although expected cell counts were low. BMI = body mass index; FMS = Functional Movement Screen; SD = standard deviation; Q1 = first quartile; Q3 = third quartile. Bolded p-values are significant at $p < .05$.

Table S5. *Female-Only FMS Score Distribution by No Injury vs ≥1 Lower-Limb Injury Status*

FMS component	Score	Overall, n (%)	No injury, n (%)	≥1 injury, n (%)
Deep Squat	0	3 (2.2%)	1 (1.0%)	2 (6.5%)
	1	5 (3.7%)	5 (4.8%)	0 (0.0%)
	2	115 (85.2%)	88 (84.6%)	27 (87.1%)
	3	12 (8.9%)	10 (9.6%)	2 (6.5%)
Hurdle Step	0	0 (0.0%)	0 (0.0%)	0 (0.0%)
	1	7 (5.2%)	4 (3.8%)	3 (9.7%)
	2	86 (63.7%)	65 (62.5%)	21 (67.7%)
	3	42 (31.1%)	35 (33.7%)	7 (22.6%)
In-Line Lunge	0	3 (2.2%)	0 (0.0%)	3 (9.7%)
	1	7 (5.2%)	6 (5.8%)	1 (3.2%)
	2	99 (73.3%)	74 (71.2%)	25 (80.6%)
	3	26 (19.3%)	24 (23.1%)	2 (6.5%)
Active Straight Leg Raise	0	0 (0.0%)	0 (0.0%)	0 (0.0%)
	1	1 (0.7%)	1 (1.0%)	0 (0.0%)
	2	35 (25.9%)	26 (25.0%)	9 (29.0%)
	3	99 (73.3%)	77 (74.0%)	22 (71.0%)
Trunk Stability Push-Up	0	0 (0.0%)	0 (0.0%)	0 (0.0%)
	1	11 (8.1%)	7 (6.7%)	4 (12.9%)
	2	74 (54.8%)	60 (57.7%)	14 (45.2%)

FMS component	Score	Overall, n (%)	No injury, n (%)	≥1 injury, n (%)
	3	50 (37.0%)	37 (35.6%)	13 (41.9%)
Rotary Stability	0	4 (3.0%)	3 (2.9%)	1 (3.2%)
	1	4 (3.0%)	4 (3.8%)	0 (0.0%)
	2	123 (91.1%)	94 (90.4%)	29 (93.5%)
	3	4 (3.0%)	3 (2.9%)	1 (3.2%)

The table displays the distribution of Functional Movement Screen component scores among female participants by no-injury and ≥1-injury status. Values are presented as n (%). Percentages are calculated within the overall female sample and within each female injury-status group. FMS = Functional Movement Screen. Component scores range from 0 to 3, with higher scores indicating better movement performance. The female sample included 135 participants, with 104 participants in the no-injury group and 31 participants in the ≥1-injury group.

Table S6. *Adjusted Logistic Regression Models for Lower-Limb Injury Outcomes Among Female Participants*

Outcome	Model	Variable	OR	95% CI	*p*-value	AUC
≥1 injury	1	Knee count	1.08	0.63, 1.86	.783	.70
		Ankle count	1.08	0.53, 2.21	.831	
		BMI	1.13	1.02, 1.26	**.021**	
		LL injury history	2.33	0.99, 5.51	.053	
	2	Knee count	1.05	0.61, 1.83	.851	.70
		Ankle count	1.03	0.50, 2.13	.940	
		BMI	1.12	1.00, 1.25	.052	
		LL injury history	2.31	0.98, 5.46	.057	
		FMS composite score	0.90	0.71, 1.14	.379	
	3	Knee count	0.97	0.53, 1.77	.914	.72
		Ankle count	1.00	0.47, 2.14	.992	
		BMI	1.11	0.99, 1.24	.067	
		LL injury history	1.94	0.78, 4.81	.154	
		Deep Squat	0.73	0.27, 1.98	.533	

Outcome	Model	Variable	OR	95% CI	*p*-value	AUC
		Hurdle Step	0.86	0.37, 2.01	.729	
		In-Line Lunge	0.40	0.16, 1.01	.053	
		Active Straight Leg Raise	1.13	0.43, 2.95	.803	
		Trunk Stability Push-Up	1.25	0.61, 2.57	.548	
		Rotary Stability	1.99	0.61, 6.48	.251	
≥2 injuries	1	Knee count	1.16	0.58, 2.29	.673	.66
		Ankle count	0.74	0.26, 2.11	.575	
		BMI	1.05	0.91, 1.20	.507	
		LL injury history	3.38	1.01, 11.35	**.049**	
	2	Knee count	1.12	0.55, 2.28	.753	.71
		Ankle count	0.62	0.21, 1.81	.385	
		BMI	1.01	0.87, 1.16	.936	
		LL injury history	3.33	0.97, 11.43	.056	
		FMS composite score	0.74	0.55, 1.00	.052	
	3	Knee count	1.05	0.47, 2.34	.902	.77
		Ankle count	0.55	0.16, 1.85	.331	
		BMI	0.99	0.84, 1.16	.863	
		LL injury history	3.45	0.92, 12.91	.066	
		Deep Squat	0.29	0.09, 0.91	**.035**	
		Hurdle Step	0.62	0.19, 2.05	.433	
		In-Line Lunge	0.49	0.15, 1.55	.225	
		Active Straight Leg Raise	0.60	0.19, 1.92	.390	
		Trunk Stability Push-Up	1.06	0.41, 2.69	.908	
		Rotary Stability	2.42	0.54, 10.90	.251	

This analysis was restricted to female participants ($N = 135$). Model 1 included knee instability count, ankle instability count, BMI, and prior lower-limb injury history. Model 2 added the FMS composite score. Model 3 replaced the FMS composite score with individual FMS component scores. Odds ratios for knee and ankle instability counts represent the change in odds associated with

each additional positive instability finding. Odds ratios for FMS component scores represent the change in odds associated with a one-point increase in the respective FMS component score. OR = odds ratio; CI = confidence interval; AUC = area under the receiver operating characteristic curve; BMI = body mass index; LL = lower limb; FMS = Functional Movement Screen. Bolded p-values are significant at $p < .05$. These sex-stratified models, particularly the ≥2-injury and Model 3 analyses, should be interpreted cautiously because of the small number of outcome events relative to the number of variables.

Table S7. *Adjusted Logistic Regression Models for Lower-Limb Injury Outcomes Among Female Participants using Binary Instability Variables (N = 135)*

Outcome	Model	Variable	OR	95% CI	p-value	AUC
≥1 injury	1	Knee instability (any)	0.91	0.33, 2.47	.848	0.70
		Ankle instability (any)	0.95	0.36, 2.52	.912	
		BMI	1.13	1.02, 1.26	**.022**	
		LL injury history	2.40	1.01, 5.70	**.047**	
	2	Knee instability (any)	0.87	0.32, 2.38	.784	0.70
		Ankle instability (any)	0.91	0.34, 2.45	.854	
		BMI	1.11	1.00, 1.24	.057	
		LL injury history	2.36	0.99, 5.62	.052	
		FMS composite score	0.89	0.71, 1.13	.335	
	3	Knee instability (any)	0.68	0.22, 2.10	.499	0.73
		Ankle instability (any)	0.87	0.31, 2.43	.783	
		BMI	1.11	0.99, 1.24	.073	
		LL injury history	1.95	0.78, 4.87	.151	
		Deep Squat	0.74	0.28, 1.99	.554	
		Hurdle Step	0.86	0.36, 2.01	.719	
		In-Line Lunge	0.37	0.14, 0.95	**.039**	
		Active Straight Leg Raise	1.11	0.43, 2.87	.837	
		Trunk Stability Push-Up	1.25	0.61, 2.56	.546	
		Rotary Stability	1.91	0.60, 6.05	.271	
≥2 injuries	1	Knee instability (any)	1.17	0.33, 4.10	.809	0.67
		Ankle instability (any)	0.60	0.15, 2.37	.464	
		BMI	1.05	0.91, 1.20	.521	
		LL injury history	3.44	1.02, 11.61	**.046**	
	2	Knee instability (any)	1.09	0.30, 3.96	.897	0.71

Outcome	Model	Variable	OR	95% CI	p-value	AUC
		Ankle instability (any)	0.52	0.13, 2.17	.373	
		BMI	1.00	0.87, 1.16	.954	
		LL injury history	3.34	0.97, 11.46	.055	
		FMS composite score	0.75	0.55, 1.01	.056	
	3	Knee instability (any)	1.00	0.24, 4.25	.996	0.77
		Ankle instability (any)	0.49	0.11, 2.29	.367	
		BMI	0.99	0.84, 1.16	.863	
		LL injury history	3.35	0.91, 12.39	.070	
		Deep Squat	0.31	0.09, 0.99	**.048**	
		Hurdle Step	0.62	0.19, 2.05	.434	
		In-Line Lunge	0.49	0.15, 1.58	.235	
		Active Straight Leg Raise	0.60	0.19, 1.91	.386	
		Trunk Stability Push-Up	1.10	0.43, 2.80	.849	
		Rotary Stability	2.31	0.51, 10.43	.277	

Model 1 included knee instability status, ankle instability status, BMI, and prior lower-limb injury history. Model 2 added the FMS composite score. Model 3 replaced the FMS composite score with individual FMS component scores. OR = odds ratio; CI = confidence interval; AUC = area under the receiver operating characteristic curve; BMI = body mass index; LL = lower limb; FMS = Functional Movement Screen. Bolded p-values are significant at $p < .05$.

Table S8. *Adjusted Logistic Regression Models for Lower-Limb Injury Outcomes Among Participants Without Prior Lower-Limb Injury History Using Binary Instability Variables (N = 210)*

Outcome	Model	Variable	OR	95% CI	p-value	AUC
≥1 injury	1	Knee instability (any)	0.42	0.12, 1.50	.182	0.61
		Ankle instability (any)	1.65	0.63, 4.30	.306	
		BMI	1.05	0.98, 1.14	.177	
	2	Knee instability (any)	0.40	0.11, 1.45	.166	0.62
		Ankle instability (any)	1.65	0.63, 4.32	.304	
		BMI	1.04	0.96, 1.13	.307	
		FMS composite score	0.93	0.76, 1.15	.521	
	3	Knee instability (any)	0.31	0.08, 1.23	.096	0.70
		Ankle instability (any)	1.76	0.65, 4.78	.264	
		BMI	1.04	0.95, 1.14	.364	

Outcome	Model	Variable	OR	95% CI	p-value	AUC
		Deep Squat	1.70	0.79, 3.69	.176	
		Hurdle Step	0.93	0.44, 1.98	.857	
		In-Line Lunge	0.30	0.10, 0.90	**.031**	
		Active Straight Leg Raise	0.87	0.45, 1.66	.668	
		Trunk Stability Push-Up	1.24	0.67, 2.30	.489	
		Rotary Stability	3.34	0.65, 17.23	.149	
≥2 injuries	1	Knee instability (any)	0.70	0.14, 3.39	.657	0.63
		Ankle instability (any)	2.15	0.62, 7.49	.228	
		BMI	1.09	0.98, 1.20	.107	
	2	Knee instability (any)	0.66	0.13, 3.23	.606	0.66
		Ankle instability (any)	2.15	0.62, 7.50	.229	
		BMI	1.07	0.96, 1.20	.230	
		FMS composite score	0.89	0.67, 1.18	.431	
	3	Knee instability (any)	0.57	0.10, 3.06	.510	0.73
		Ankle instability (any)	2.50	0.68, 9.14	.166	
		BMI	1.07	0.95, 1.20	.292	
		Deep Squat	0.92	0.32, 2.62	.878	
		Hurdle Step	1.24	0.43, 3.56	.690	
		In-Line Lunge	0.45	0.10, 1.90	.275	
		Active Straight Leg Raise	0.51	0.22, 1.21	.126	
		Trunk Stability Push-Up	1.46	0.63, 3.41	.381	
		Rotary Stability	3.07	0.35, 27.05	.311	

Model 1 included knee instability status, ankle instability status, and BMI. Model 2 added the FMS composite score. Model 3 replaced the FMS composite score with individual FMS component scores. No-history models excluded prior lower-limb injury history. OR = odds ratio; CI = confidence interval; AUC = area under the receiver operating characteristic curve; BMI = body mass index; FMS = Functional Movement Screen. Bolded p-values are significant at $p < .05$.

Table S9. *Adjusted Logistic Regression Models for Lower-Limb Injury Outcomes Among Female Participants Without Prior Lower-Limb Injury History*

Outcome	Model	Variable	OR	95% CI	p-value	AUC
≥1 injury	1	Knee count	1.34	0.59, 3.07	.486	.69
		Ankle count	1.75	0.57, 5.35	.327	
		BMI	1.25	1.05, 1.47	**.010**	
	2	Knee count	1.30	0.56, 3.01	.548	.71
		Ankle count	1.71	0.55, 5.30	.355	
		BMI	1.23	1.03, 1.47	**.024**	
		FMS composite score	0.91	0.59, 1.41	.671	
	3	Knee count	1.44	0.56, 3.71	.447	.80
		Ankle count	1.97	0.55, 7.09	.298	
		BMI	1.32	1.06, 1.66	**.014**	
		Deep Squat	0.72	0.16, 3.23	.671	
		Hurdle Step	0.59	0.13, 2.59	.481	
		In-Line Lunge	0.51	0.08, 3.38	.489	
		Active Straight Leg Raise	4.37	0.37, 50.99	.240	
		Trunk Stability Push-Up	1.51	0.43, 5.36	.520	
		Rotary Stability	11.43	0.45, 287.27	.139	
≥2 injuries	1	Model failed	—	—	—	—
	2	Knee count	1.17	0.29, 4.72	.829	.83
		Ankle count	0.00	0.00, ∞	—	
		BMI	1.14	0.88, 1.48	.327	
		FMS composite score	0.64	0.33, 1.26	.199	
	3	Knee count	0.70	0.09, 5.54	.738	.88
		Ankle count	0.00	0.00, ∞	—	
		BMI	1.26	0.84, 1.89	.260	

Outcome	Model	Variable	OR	95% CI	p-value	AUC
		Deep Squat	0.11	0.01, 1.20	.070	
		Hurdle Step	0.45	0.05, 3.68	.455	
		In-Line Lunge	2.24	0.14, 36.19	.571	
		Active Straight Leg Raise	0.29	0.01, 6.16	.426	
		Trunk Stability Push-Up	1.13	0.15, 8.43	.904	
		Rotary Stability	1.50	0.00, 607.34	.895	

This analysis was restricted to female participants without prior lower-limb injury history (*N* = 69). Model 1 included knee instability count, ankle instability count, and BMI. Model 2 added the FMS composite score. Model 3 replaced the FMS composite score with individual FMS component scores. Odds ratios for knee and ankle instability counts represent the change in odds associated with each additional positive instability finding. Odds ratios for FMS component scores represent the change in odds associated with a one-point increase in the respective FMS component score. OR = odds ratio; CI = confidence interval; AUC = area under the receiver operating characteristic curve; BMI = body mass index; FMS = Functional Movement Screen. Bolded p-values are significant at $p < .05$. The ≥2-injury models should be interpreted with extreme caution because only 4 events occurred in this subgroup; the Model 1 ≥2-injury model failed, and some estimates showed evidence of instability or quasi-separation, including infinite confidence limits.

Table S10. *Adjusted Logistic Regression Models for Lower-Limb Injury Outcomes Among Female Participants Without Prior Lower-Limb Injury History Using Binary Instability Variables (N = 69)*

Outcome	Model	Variable	OR	95% CI	p-value	AUC
≥1 injury	1	Knee instability (any)	0.82	0.14, 4.97	.834	0.71
		Ankle instability (any)	1.18	0.19, 7.20	.860	
		BMI	1.23	1.04, 1.46	**.014**	
	2	Knee instability (any)	0.74	0.12, 4.53	.740	0.76
		Ankle instability (any)	1.10	0.17, 6.93	.923	
		BMI	1.21	1.02, 1.44	**.033**	
		FMS composite score	0.87	0.57, 1.32	.512	
	3	Knee instability (any)	0.68	0.08, 5.64	.724	0.80
		Ankle instability (any)	1.55	0.20, 12.02	.677	
		BMI	1.29	1.04, 1.61	**.023**	
		Deep Squat	0.74	0.17, 3.19	.683	

Outcome	Model	Variable	OR	95% CI	p-value	AUC
		Hurdle Step	0.56	0.12, 2.58	.459	
		In-Line Lunge	0.42	0.06, 2.72	.362	
		Active Straight Leg Raise	3.15	0.34, 29.21	.313	
		Trunk Stability Push-Up	1.36	0.41, 4.56	.618	
		Rotary Stability	10.29	0.37, 286.08	.169	
≥2 injuries	1	Knee instability (any)	1.13	0.10, 13.07	.921	0.70
		Ankle instability (any)	0.00	0.00, ∞	—	
		BMI	1.18	0.92, 1.50	.191	
	2	Knee instability (any)	0.79	0.06, 9.94	.856	0.84
		Ankle instability (any)	0.00	0.00, ∞	—	
		BMI	1.15	0.88, 1.50	.314	
		FMS composite score	0.63	0.32, 1.25	.186	
	3	Knee instability (any)	0.26	0.01, 12.34	.494	0.89
		Ankle instability (any)	0.00	0.00, ∞	—	
		BMI	1.31	0.85, 2.03	.219	
		Deep Squat	0.11	0.01, 1.24	.074	
		Hurdle Step	0.39	0.04, 3.41	.394	
		In-Line Lunge	2.45	0.14, 43.83	.542	
		Active Straight Leg Raise	0.23	0.01, 5.12	.350	
		Trunk Stability Push-Up	1.27	0.17, 9.30	.814	
		Rotary Stability	1.57	0.00, 766.44	.886	

Model 1 included knee instability status, ankle instability status, and BMI. Model 2 added the FMS composite score. Model 3 replaced the FMS composite score with individual FMS component scores. No-history models excluded prior lower-limb injury history. OR = odds ratio; CI = confidence interval; AUC = area under the receiver operating characteristic curve; BMI = body mass index; FMS = Functional Movement Screen. Bolded p-values are significant at $p < .05$.

Table S11. *Adjusted Logistic Regression Models for Lower-Limb Injury Outcomes Among Male Participants Without Prior Lower-Limb Injury History Using Binary Instability Variables (N = 141)*

Outcome	Model	Variable	OR	95% CI	p-value	AUC
≥1 injury	1	Knee instability (any)	0.17	0.02, 1.41	.101	0.59
		Ankle instability (any)	2.10	0.64, 6.87	.219	
		BMI	1.00	0.91, 1.10	.948	
	2	Knee instability (any)	0.17	0.02, 1.39	.097	0.60
		Ankle instability (any)	2.13	0.65, 6.97	.212	
		BMI	1.00	0.90, 1.11	.959	
		FMS composite score	0.96	0.74, 1.23	.735	
	3	Knee instability (any)	0.14	0.01, 1.40	.095	0.75
		Ankle instability (any)	2.20	0.63, 7.60	.215	
		BMI	0.98	0.88, 1.10	.789	
		Deep Squat	2.30	0.93, 5.72	.072	
		Hurdle Step	1.05	0.40, 2.75	.926	
		In-Line Lunge	0.17	0.03, 0.87	**.033**	
		Active Straight Leg Raise	0.55	0.25, 1.20	.133	
		Trunk Stability Push-Up	1.79	0.72, 4.46	.209	
		Rotary Stability	3.43	0.40, 29.55	.262	
≥2 injuries	1	Knee instability (any)	0.35	0.04, 3.17	.348	0.66
		Ankle instability (any)	4.22	1.03, 17.30	**.045**	
		BMI	1.07	0.94, 1.21	.302	
	2	Knee instability (any)	0.34	0.04, 3.13	.339	0.66
		Ankle instability (any)	4.27	1.04, 17.54	**.044**	
		BMI	1.06	0.93, 1.21	.381	
		FMS composite score	0.95	0.69, 1.33	.782	
	3	Knee instability (any)	0.32	0.03, 3.60	.358	0.78
		Ankle instability (any)	4.46	1.03, 19.32	**.046**	
		BMI	1.04	0.90, 1.20	.582	
		Deep Squat	1.73	0.52, 5.75	.370	
		Hurdle Step	1.83	0.50, 6.67	.361	
		In-Line Lunge	0.30	0.04, 2.10	.224	
		Active Straight Leg Raise	0.45	0.16, 1.26	.130	

Outcome	Model	Variable	OR	95% CI	p-value	AUC
		Trunk Stability Push-Up	1.35	0.45, 4.10	.593	
		Rotary Stability	2.00	0.14, 28.18	.608	

Model 1 included knee instability status, ankle instability status, and BMI. Model 2 added the FMS composite score. Model 3 replaced the FMS composite score with individual FMS component scores. No-history models excluded prior lower-limb injury history. OR = odds ratio; CI = confidence interval; AUC = area under the receiver operating characteristic curve; BMI = body mass index; FMS = Functional Movement Screen. Bolded p-values are significant at $p < .05$.

Table S12. *Sensitivity Analysis: Adjusted Logistic Regression Models for ≥2 Lower-Limb Injuries in the Full Sample After Excluding Sports with Zero Events Using Binary Instability Variables*

Outcome	Model	Variable	OR	95% CI	p-value	AUC
≥2 injuries	1	Knee instability (any)	0.73	0.29, 1.88	.515	0.63
		Ankle instability (any)	1.38	0.62, 3.07	.427	
		BMI	1.02	0.95, 1.09	.538	
		LL injury history	2.16	1.10, 4.25	**.026**	
	2	Knee instability (any)	0.69	0.27, 1.79	.445	0.66
		Ankle instability (any)	1.34	0.60, 3.01	.474	
		BMI	1.00	0.93, 1.07	.982	
		LL injury history	2.16	1.10, 4.27	**.026**	
		FMS composite score	0.83	0.70, 1.00	**.047**	
	3	Knee instability (any)	0.62	0.23, 1.66	.338	0.68
		Ankle instability (any)	1.30	0.57, 2.99	.531	
		BMI	1.00	0.92, 1.07	.933	
		LL injury history	2.07	1.03, 4.15	**.041**	
		Deep Squat	0.75	0.40, 1.40	.362	
		Hurdle Step	1.35	0.70, 2.60	.367	
		In-Line Lunge	0.48	0.23, 1.02	.055	
		Active Straight Leg Raise	0.61	0.34, 1.10	.101	
		Trunk Stability Push-Up	1.03	0.58, 1.84	.918	
		Rotary Stability	0.83	0.36, 1.92	.661	

This sensitivity analysis excluded sports with zero ≥2-injury events (N = 333; events = 41; non-events = 292). Model 1 included any knee instability, any ankle instability, BMI, and prior lower-limb injury history. Model 2 added the FMS composite score. Model 3

replaced the FMS composite score with individual FMS component scores. Odds ratios for binary instability variables compare participants with any instability to those without instability. Odds ratios for FMS component scores represent the change in odds associated with a one-point increase in the respective component score. OR = odds ratio; CI = confidence interval; AUC = area under the receiver operating characteristic curve; BMI = body mass index; LL = lower limb; FMS = Functional Movement Screen. Bolded p-values are significant at $p < .05$. Model 3 should be interpreted cautiously because of the increased number of variables relative to the number of events.

Table S13. *Sensitivity Analysis: Adjusted Logistic Regression Models for ≥2 Lower-Limb Injuries Among Female Participants After Excluding Zero-Event Sports*

Outcome	Model	Variable	OR	95% CI	p-value	AUC
≥2 injuries	1	Knee count	1.03	0.50, 2.09	.940	0.72
		Ankle count	0.79	0.27, 2.31	.674	
		BMI	1.11	0.95, 1.30	.182	
		LL injury history	3.93	1.14, 13.58	**.030**	
	2	Knee count	0.95	0.44, 2.05	.891	0.76
		Ankle count	0.51	0.15, 1.68	.266	
		BMI	1.04	0.88, 1.23	.621	
		LL injury history	4.35	1.19, 15.95	**.027**	
		FMS composite score	0.64	0.45, 0.92	**.016**	
	3	Knee count	0.72	0.28, 1.89	.507	0.84
		Ankle count	0.35	0.08, 1.52	.159	
		BMI	1.01	0.83, 1.22	.935	
		LL injury history	5.88	1.23, 28.07	**.026**	
		Deep Squat	0.20	0.04, 0.95	**.043**	
		Hurdle Step	0.79	0.20, 3.06	.732	
		In-Line Lunge	0.34	0.08, 1.50	.156	
		Active Straight Leg Raise	0.22	0.05, 1.01	.051	
		Trunk Stability Push-Up	0.66	0.21, 2.06	.471	
		Rotary Stability	1.32	0.30, 5.79	.714	

This sensitivity analysis excluded sports with zero ≥2-injury events and included 107 participants (15 with ≥2 injuries and 92 with <2 injuries). Model 1 included knee instability count, ankle instability count, BMI, and prior lower-limb injury history. Model 2 added the FMS composite score. Model 3 replaced the FMS composite score with individual FMS component scores. OR = odds ratio; CI = confidence interval; AUC = area under the receiver operating characteristic curve; BMI = body mass index; LL = lower

limb; FMS = Functional Movement Screen. Bolded p-values are significant at p < .05. Model 3 should be interpreted cautiously because of the increased number of variables relative to the number of outcome events.

Table S14. *Sensitivity Analysis: Adjusted Logistic Regression Models for ≥2 Lower-Limb Injuries Among Female Participants After Excluding Sports with Zero Events Using Binary Instability Variables*

Outcome	Model	Variable	OR	95% CI	p-value	AUC
≥2 injuries	1	Knee instability (any)	0.94	0.26, 3.43	.930	0.72
		Ankle instability (any)	0.64	0.16, 2.62	.538	
		BMI	1.11	0.95, 1.30	.189	
		LL injury history	4.05	1.17, 14.05	**.028**	
	2	Knee instability (any)	0.79	0.20, 3.14	.739	0.76
		Ankle instability (any)	0.41	0.09, 1.95	.263	
		BMI	1.04	0.88, 1.23	.613	
		LL injury history	4.41	1.21, 16.11	**.025**	
		FMS composite score	0.65	0.46, 0.93	**.017**	
	3	Knee instability (any)	0.52	0.10, 2.74	.439	0.84
		Ankle instability (any)	0.30	0.05, 1.77	.184	
		BMI	1.01	0.84, 1.22	.882	
		LL injury history	5.62	1.22, 25.95	**.027**	
		Deep Squat	0.23	0.05, 1.06	.060	
		Hurdle Step	0.81	0.21, 3.14	.763	
		In-Line Lunge	0.35	0.08, 1.56	.169	
		Active Straight Leg Raise	0.23	0.05, 1.01	.052	
		Trunk Stability Push-Up	0.69	0.22, 2.17	.526	
		Rotary Stability	1.21	0.28, 5.19	.802	

This sensitivity analysis excluded sports with zero ≥2-injury events and included 107 participants (15 with ≥2 injuries and 92 with <2 injuries). Model 1 included any knee instability, any ankle instability, BMI, and prior lower-limb injury history. Model 2 added the FMS composite score. Model 3 replaced the FMS composite score with individual FMS component scores. Odds ratios for binary instability variables compare participants with any instability to those without instability. Odds ratios for FMS component scores represent the change in odds associated with a one-point increase in the respective component score. OR = odds ratio; CI = confidence interval; AUC = area under the receiver operating characteristic curve; BMI = body mass index; LL = lower limb; FMS =

Functional Movement Screen. Bolded p-values are significant at p < .05. Model 3 should be interpreted cautiously because of the increased number of variables relative to the number of events.

Table S15. *Sensitivity Analysis: Adjusted Logistic Regression Models for ≥2 Lower-Limb Injuries Among Male Participants Without Prior Lower-Limb Injury History After Excluding Zero-Event Sports*

Outcome	Model	Variable	OR	95% CI	p-value	AUC
≥2 injuries	1	Knee count	0.67	0.31, 1.44	.305	0.66
		Ankle count	3.39	1.10, 10.41	**.033**	
		BMI	1.04	0.92, 1.19	.522	
	2	Knee count	0.66	0.30, 1.45	.299	0.66
		Ankle count	3.43	1.11, 10.62	**.033**	
		BMI	1.04	0.90, 1.19	.603	
		FMS composite score	0.96	0.69, 1.35	.833	
	3	Knee count	0.70	0.32, 1.54	.370	0.78
		Ankle count	3.25	1.02, 10.37	**.047**	
		BMI	1.03	0.88, 1.19	.739	
		Deep Squat	1.48	0.41, 5.31	.544	
		Hurdle Step	1.81	0.50, 6.51	.366	
		In-Line Lunge	0.34	0.05, 2.31	.269	
		Active Straight Leg Raise	0.48	0.18, 1.31	.152	
		Trunk Stability Push-Up	1.58	0.46, 5.43	.465	
		Rotary Stability	1.77	0.17, 19.00	.635	

This sensitivity analysis excluded sports with zero ≥2-injury events and was restricted to male participants without prior lower-limb injury history (N = 118; 12 with ≥2 injuries and 106 with <2 injuries). Model 1 included knee instability count, ankle instability count, and BMI. Model 2 added the FMS composite score. Model 3 replaced the FMS composite score with individual FMS component scores. OR = odds ratio; CI = confidence interval; AUC = area under the receiver operating characteristic curve; BMI = body mass index; FMS = Functional Movement Screen. Bolded p-values are significant at p < .05. Model 3 should be interpreted cautiously because of the increased number of variables relative to the number of outcome events.

Table S16. *Sensitivity Analysis: Adjusted Logistic Regression Models for ≥2 Lower-Limb Injuries Among Male Participants Without Prior Lower-Limb Injury History After Excluding Sports with Zero Events Using Binary Instability Variables*

Outcome	Model	Variable	OR	95% CI	p-value	AUC
≥2 injuries	1	Knee instability (any)	0.21	0.02, 2.18	.189	0.66
		Ankle instability (any)	7.72	1.62, 36.79	**.010**	
		BMI	1.03	0.91, 1.18	.620	
	2	Knee instability (any)	0.19	0.02, 2.10	.176	0.67
		Ankle instability (any)	8.04	1.66, 38.91	**.010**	
		BMI	1.02	0.88, 1.18	.772	
		FMS composite score	0.93	0.66, 1.29	.648	
	3	Knee instability (any)	0.17	0.01, 2.31	.182	0.80
		Ankle instability (any)	7.93	1.53, 40.99	**.013**	
		BMI	1.00	0.85, 1.17	.984	
		Deep Squat	1.40	0.38, 5.11	.610	
		Hurdle Step	2.20	0.57, 8.52	.255	
		In-Line Lunge	0.30	0.04, 2.21	.238	
		Active Straight Leg Raise	0.45	0.16, 1.29	.139	
		Trunk Stability Push-Up	1.21	0.37, 3.91	.754	
		Rotary Stability	1.62	0.14, 19.24	.704	

This sensitivity analysis excluded sports with zero ≥2-injury events and was restricted to male participants without prior lower-limb injury history (N = 118; 12 with ≥2 injuries and 106 with <2 injuries). Model 1 included any knee instability, any ankle instability, BMI, and prior lower-limb injury history when applicable. Model 2 added the FMS composite score. Model 3 replaced the FMS composite score with individual FMS component scores. Odds ratios for binary instability variables compare participants with any instability to those without instability. Odds ratios for FMS component scores represent the change in odds associated with a one-point increase in the respective component score. OR = odds ratio; CI = confidence interval; AUC = area under the receiver operating characteristic curve; BMI = body mass index; FMS = Functional Movement Screen. Bolded p-values indicate statistical significance at $p < .05$. Model 3 should be interpreted cautiously because of the increased number of variables relative to the number of events.
